# Changes in Serum Amyloid A (SAA) Concentration in Arabian Endurance Horses During First Training Season

**DOI:** 10.3390/ani9060330

**Published:** 2019-06-08

**Authors:** Olga Witkowska-Piłaszewicz, Piotr Bąska, Michał Czopowicz, Magdalena Żmigrodzka, Jarosław Szczepaniak, Ewa Szarska, Anna Winnicka, Anna Cywińska

**Affiliations:** 1 Department of Pathology and Veterinary Diagnostics, Faculty of Veterinary Medicine, Warsaw University of Life Sciences, 02-787 Warsaw, Poland; mzmigro@gmial.com (M.Ż.); anna_winnicka@sggw.pl (A.W.); 2 Division of Pharmacology and Toxicology, Department of Preclinical Sciences, Faculty of Veterinary Medicine, Warsaw University of Life Sciences, 02-787 Warsaw, Poland; piotr_baska@sggw.pl; 3 Laboratory of Veterinary Epidemiology and Economics, Faculty of Veterinary Medicine, Warsaw University of Life Sciences, 02-787 Warsaw, Poland; michal_czopowicz@sggw.pl; 4 Department of Animal Nutrition and Biotechnology, Faculty of Animal Sciences, Warsaw University of Life Sciences, 02-787 Warsaw, Poland; jaroslaw_szczepaniak@sggw.pl; 5 Military Institute of Hygiene and Epidemiology, 01-163 Warsaw, Poland; eszarska@gmail.com

**Keywords:** APPs, exercise-induced APR, sport medicine, equine, hematology

## Abstract

Sport training leads to adaptation to physical effort that is reflected by the changes in blood parameters. In equine endurance athletes, blood testing is accepted as a support in training, however, only the changes before versus after exercise in creatine phosphokinase activity (CPK) and basic blood parameters are usually measured. This study is the first longitudinal investigation of the changes in routinely measured blood parameters and, additionally, serum amyloid A (SAA), during seven months, in Arabian horses introduced to endurance training and competing in events for young horses. It has been determined that CPK, aspartate aminotransferase (AST), packed cell volume (PCV), hemoglobin concentration, red blood cell count (RBC), and concentration of total serum protein (TSP) slightly increased after training sessions and competitions in similar manner. The increase in white blood cell (WBC) count was higher after competitions and SAA increased only after competitions. Total protein concentration was the only parameter that increased with training during a 7-month program. SAA indicated only in the case of heavy effort, and, it thus may be helpful in the monitoring of training in young horses. In an optimal program, its concentration should not increase after a training session but only after heavy effort, which should not be repeated too often.

## 1. Introduction

Optimal training is crucial for animal welfare in order to produce exercise adaptation but also to maintain good health in a horse. The analysis of change in routine blood parameters is used for the evaluation of training progress, as well as general health, but does not provide the information necessary for estimating subclinical disorders that may worsen with effort and result in interruption of trainings or even termination of sport career. Therefore, additional parameters, such as serum amyloid A (SAA), have been considered. The aim of this longitudinal study was to investigate the changes in SAA concentration in Arabian endurance horses during their first training season, with special regard to the relationship between SAA concentration and the time and type of effort (training versus competition). Our study indicates that, in the horses, the measurements of SAA concentration together with commonly accepted parameters give additional insight into health control of the horse.

Endurance riding is an equestrian sport, involving completing the distances from 40 km to 160 km, depending on the age and sport level of horses, confirmed by previous finishing competitions required by the regulations. All distances are divided into loops, 20 km to 40 km each, with a time check and obligatory veterinary inspection (vet gate), in order to detect if the horses are fit to continue the ride. Horses with irregular gait or metabolic abnormalities are eliminated from competitions.

Horses can reach the highest sport level (160 km competitions) after several years of training and competing at shorter distances since the career of endurance horses spans many years. Not only the time of finishing the ride, but mostly points granted on the veterinary health check, matter for the final score. Therefore, careful regular evaluation of health condition of endurance horses is crucial. While during the competition it is based solely on physical examination performed by a veterinarian, during training it is supported by periodical monitoring of blood parameters. In endurance horses, creatine phosphokinase activity (CPK) poses the most important functional measurement and, additionally, aspartate aminotransferase (AST), packed cell volume (PCV), hemoglobin concentration (HGB), red blood cell count (RBC), and concentration of total serum protein (TSP), glucose, phosphorus, and potassium [1,2] are commonly determined. The analysis of change in these parameters over time provides information on metabolic alterations associated with training progress, however, it does not allow to detect developing disorders at the subclinical stage [3]. Therefore, additional parameters characterizing the acute phase response (APR) have been considered [4,5,6].

Typical APR that occurs in inflammation involves marked increases in the concentrations of pro-inflammatory cytokines and acute phase proteins (APPs), such as SAA, which is the major APP in horses [7,8,9], C-reactive protein, and haptoglobin. A similar reaction has been described in humans [10,11] and animals [12,13] after strenuous endurance events, however, certain differences between disease-induced and exercise-induced APR exist. In horses, the latter is characterized only by the increases in the concentrations of some cytokines [14] and SAA, with other APPs remaining unchanged [12]. Such reactions may also occur during training [15], but its role is yet to be clarified.

Therefore, the aim of this longitudinal study was to investigate the changes in SAA concentration in Arabian endurance horses during their first training season, with special regard to the relationship between SAA concentration and the time and type of effort (training versus competition).

## 2. Materials and Methods

### 2.1. Horses and Blood Sampling

Eight privately-owned, healthy, 6–7 years old, previously untrained Arabian horses (two mares and six geldings) that were starting their endurance training and sport carrier were enrolled in this study. The horses were housed in two stables in similar conditions, fed, and trained according to similar protocols. All exercises were provided under similar terrain conditions. The training involved daily sessions with the exercise-load depending on the horse’s condition and increasing with time; altogether the horses covered about 250 km per month, and the sessions with high exercise-load were performed every 14–20 days. Beginning with third month of training, the horses were introduced to endurance competitions at limited distance. The animals were monitored for the whole training season (the first training season in their carrier) and examined before and after training sessions selected for the study—the ones with high exercise-load (25–28 km, speed 14–15 km/h) and before and after finishing the competitions (42–45 km, speed 14–16 km/h). Standard clinical examination performed before and after each training session included in the study revealed no clinical symptoms of disease. The study covered seven months and included five training sessions and three competitions, so the maximal number of examinations for individual horse was eight; however, horses entered a different number of competitions.

Blood samples were collected before and within one h after the training sessions or competitions during seven months of training. All samples were obtained during standard veterinary diagnostic procedures; thus, according Polish law, the approval by Local Commission for Ethics in Animal Experiments was not required. All blood samples were acquired by a jugular venipuncture using a BD Vacutainer system into K2-EDTA tubes for hematological tests and plain tubes for serum analyses.

### 2.2. Haematology and Serum Analysis

EDTA blood samples were kept at + 4 °C and examined within 5 h for hematological parameters: white blood cell (WBC) count, PCV, hemoglobin concentration, and RBC in an automated analyzer calibrated for equine species (ABC Vet, Horiba ABX).

The dry tubes were centrifuged (4380× *g*, 5 min), and serum free from any apparent hemolysis was aspirated for further analyses. AST and CPK activity were determined using automated clinical biochemistry analyzer (Miura One, ISE. S.r.l., Albuccione, Italy). TSP concentration was measured by refractometer technique (Reichert Rhino Vet 360, Munich, Germany). For all measurements, Pointe Scientific (Canton, USA) reagents, standards, calibrators, and controls were used.

SAA concentrations were measured using immunoenzymatic commercial assay (PHASE SAA Assay, Tridelta Ltd., Maynooth, Ireland). Sample dilution was 1:1000 instead of 1:2000, recommended by the manufacturer’s protocol, and the results were appropriately recalculated. The assay, including the dilution, was previously validated for determination of SAA concentrations in horses [12]. The absorbance was measured by Multiscan Reader (Labsystem, Helsinki, Finland) using a Genesis V 3.00 software program.

To avoid the influence of hemoconcentration, SAA concentration was adjusted by TSP, according to the formula:

SAA_adj_ = SAA × TSP_0_/TSP_1_. (1)

SAA is the SAA concentration, TSP_0_ is the TSP level before exertion, and TSP_1_ is the total serum protein level after exertion.

### 2.3. Statistical Analysis

Numerical variables were presented as the arithmetic mean ± standard deviation (SD) or median and interquartile range (IQR), unless normally distributed. The range was reported in all cases. The influence of two different types of effort (training and competition) on blood parameters was assessed using the hierarchical linear model controlling for potential confounding factors: the individual effect of a horse (included since horses were examined a different number of times), the number of efforts the horse had undergone before, and the time of blood collection (before and after the effort). The interaction between the time of blood collection and the type of effort was entered in the last step of developing the model and retained only when significant. Otherwise standardized regression coefficients (β) and *p*-values for the model without interaction were interpreted. Pairwise comparisons were performed only when a given factor (i.e., number of efforts, time of blood collection, or type of effort) proved significant in the hierarchical model on measurements averaged for an individual horse with respect to these factors, which proved insignificant in the hierarchical model. The paired-sample Student’s *t*-test, or the Wilcoxon signed rank test and sign test in the case of non-normally distributed variables, were used in pairwise comparisons. A significance level (α) was set at 0.05. Statistical analysis was performed in IBM SPSS Statistics 24 (Microsoft, New York, NY, USA) and TIBCO Statistica 13.3.0 (TIBCO Software Inc., Palo Alto, CA, USA).

## 3. Results

In all horses hematological and serum biochemical parameters determined before the physical effort fell within relevant reference intervals [2]. During the 7-month training season, TSP was the only blood parameter that significantly increased, while the rest remained unaffected by the number of efforts the horse had undergone (Table 1).

**Figure 1:**
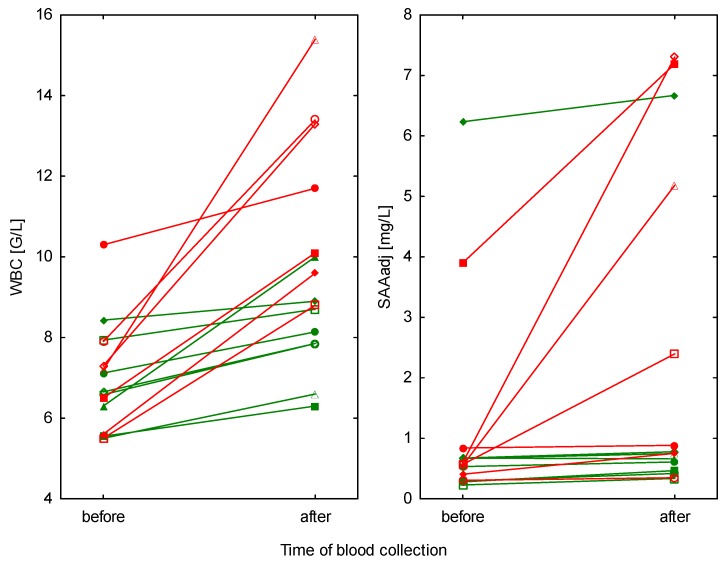
WBC counts and SAA concentrations before and after training sessions in eight horses (green lines) and before and after competitions in seven horses (red lines).

The significant interaction between type of effort and time of blood collection was observed only for WBC and SAA_adj_ (Table 1)—they significantly increased after competition, whereas remained unchanged after training (Table 2, Figure 1). The highest individual SAA concentrations after competition reached 10.6 mg/L. All remaining hematological (RBC, HGB, PCV) and serum biochemical parameters (CPK, AST, TSP) slightly rose both after training and competition (Table 3).

## 4. Discussion

This is the first study presenting the changes in blood parameters of endurance horses monitored during 7 months in their first training season. As expected, the parameters routinely measured in the monitoring of endurance training (CPK, AST, RBC, HGB, and PCV) slightly increased likewise after the training and competitions.

CPK and AST activities are commonly used in horses as the indicators of any kind of muscle fatigue or damage. After strenuous exercise, they increase from four- to 35-fold and from two- to six-fold, respectively [16]. In muscle damage, they are markedly elevated [16,17] and clinical sings, such as lameness, occur. Exercise-induced elevations in CPK activity may be attenuated by conditioning [16] but do not provide information on the magnitude of muscle damage [17]. In our study, CPK and AST activities were elevated significantly but only slightly less than two-fold, regardless of the type of effort.

RBC, HGB, and PCV values are routinely examined in sport horses. In our study, the differences between resting and post-exercise measurements were similar during training and competition. Exercise-induced hematological changes result from spleen contraction and the decrease in plasma volume [18]. The latter is also likely to account for the post-exercise increase of TSP. Moreover, basal TSP concentration was the only parameter measured in our study that increased along with training process. It has been hypothesized that in human during training, plasma total osmolar and albumin contents increase to maintain constant during plasma volume expansion due to training-induced hypervolemia [19], which is consistent with our findings in horses. However, although another study observed no increase of TSP concentration after training season, this could be due to the fact that this study was almost two-fold shorter and involved different training regimens for each horse [20].

It has been hypothesized that, in inexperienced horses, physical effort during training, particularly a heavy one, induces a reaction similar to APR [15]. Several theories explaining the causes of exercise-induced APR have been proposed, however, mostly in regard to extreme effort during competitions or overtraining [21,22,23]. One of these hypotheses is that a high load of training produces muscle and/or skeletal and/or joint injuries, including microinjuries, which stimulate circulating monocytes and other cells to produce pro-inflammatory cytokines, such as tumor necrosis factor α (TNF-α), interleukin 1 (IL-1), and interleukin 6 (IL-6) [14,22]. Another widely accepted theory is that APR results from glycogen depletion in working muscles, which in turn results in IL-6 release [10,24]. IL-6 promotes pleiotropic effects, i.e., hepatic glycogenolysis and lipolysis, but also stimulates the production of APPs, including SAA in the liver [5,7,9].

Exercise-induced APR in horses has been postulated to result from an acute challenge to muscle metabolism, rather than muscle damage; however, definitive evidence is still lacking [25]. In our study, an increase of SAA concentration (up to 16-fold) was observed after competitions but not during training and never reached high values. Even the highest individual SAA concentrations after competition were still relatively low (max. 10.62 mg/L), compared with those reported in horses with acute inflammation in the course of bacterial or viral infections, where SAA concentration exceeds 100 mg/L [26,27]. In the case of exercise-induced APR, as described earlier, the highest (10-fold or more) increases in SAA concentration have been reported in endurance horses that completed the longest (120–160 km) distances [12], whereas after a moderate distance ride or strenuous training in inexperienced horses was slighter but still significant, resulting in an observed two to four-fold elevation of SAA concentration [15]. The unique feature of this response was that SAA levels tended to increase proportionally to the covered distance [28].

Our results imply that training sessions do not trigger the increase in SAA concentration when the workload is optimal to produce adaptation and maintain good health. Moderate increases of SAA after competitions indicated a heavier challenge, particularly when the distance covered two training sessions. However, the increases were not high enough to indicate pathology but, rather, the physiological response to the relatively high workload, given that the horses were in their first training season. Our previous study revealed that, in horses trained for the longest distances, SAA concentrations did not rise after moderate distance, i.e., up to 60 km long rides [12]; however, when the resting SAA concentration exceeded 1 mg/l, the horse was likely not to complete a long distance (i.e., 120 and 160 km) endurance competition [21]. It has also been suggested that in young, inexperienced endurance horses, SAA concentrations at rest are higher [15], which is consistent with the findings of our present study. These facts suggest that SAA concentrations at rest may decrease with training due to adaptational muscular remodeling in young animals and then increase only in the response to the heaviest efforts or pathology. Our study indicates that such phenomena do not occur in the first training season, so it probably requires a longer training process.

Another parameter that increased significantly only after competition was WBC. Higher WBC values are likely to be a combined result of hemoconcentration and the release of neutrophil marginal and splenic pool triggered by adrenaline, noradrenaline, and cortisol [29].

We enrolled in the study young (6–7 years old), healthy endurance Arabian horses to minimize the influence of potential confounding factors, such as aging and disease [30], but individual differences among horses still posed limitations of the study.

## 5. Conclusions

Our study indicates that, in the horses that begin their endurance carrier, the measurements of SAA concentration, together with commonly accepted parameters, give additional insight into the training process. In contrast to routine measurements, which rise in a similar manner regardless of the type of the effort, the increase in SAA concentration only indicated an excessive stress-load after heavy effort, which should not be repeated too often. Thus, in unexperienced horses in the beginning of their carrier, the moderate increases in SAA level indicate heavy effort, while higher increases can alert the veterinarian to the onset of different diseases, even without clear clinical signs.

## Figures and Tables

**Table 1:** Results of the univariate hierarchical linear model investigating the link between four independent variables (number of efforts in the season, type of effort, time of blood collection, and the interaction between type of effort and time of blood collection) and the level of hematological and serum biochemical parameters, adjusted by the individual effect of the horse.

Parameter	Unit	Individual Effect of the Horse	Number of Efforts in the Season		Time of Blood Collection (Before vs. After) ^b^		Type of Effort (Training vs. Competition) ^b^		Interaction between Type of Effort and Time of Blood Collection
		*p*-Value	Standardized Regression Coefficient (β)	*p*-Value	Standardized Regression Coefficient (β)	*p*-Value	Standardized Regression Coefficient (β)	*p*-Value	*p*-Value
WBC	10^9^/L	0.546	- ^c^	0.264	- ^c^	0.051	- ^c^	0.883	<0.001
RBC	10^12^/L	<0.001	- ^c^	0.601	0.243	0.007	- ^c^	0.388	0.550
HGB	mmol/L	<0.001	- ^c^	0.859	0.258	0.019	- ^c^	0.098	0.525
PCV	1/1	<0.001	- ^c^	0.060	0.246	0.008	- ^c^	0.930	0.584
TSP	g/L	0.177	0.412	0.007	0.371	0.002	- ^c^	0.457	0.855
AST	U/L	0.106	- ^c^	0.148	0.288	0.016	- ^c^	0.899	0.767
CPK	U/L	0.022	- ^c^	0.369	0.312	0.007	- ^c^	0.089	0.108
SAA_adj_^a^	mg/L	<0.001	- ^c^	0.169	- ^c^	0.249	- ^c^	0.717	0.045

^a^ entered into the model after logarithmic transformation; ^b^ if interaction was non-significant β and *p*-values without interaction were used; ^c^ reported only if significant (α = 0.05). Abbreviations: WBC, white blood cell count; RBC, red blood cell count; HGB, hemoglobin concentration; PCV, packed cell volume; TSP, total serum protein concentration; AST, aspartate aminotransferase activity; CPK, creatine phosphokinase activity; SAA_adj_, serum amyloid A adjusted.

**Table 2:** WBC and SAA_adj_ before and after the effort (training and competition).

Parameter Unit	Type of Effort	Time of Blood Collection		Before vs. After *p*-Value
		Before Effort	After Effort	
WBC (10^9^/L) mean ± SD (range)	training (*n* = 8)	6.8 ± 1.0 (5.5–8.4)	8.0 ± 1.2 (6.3–10.0)	0.009 ^a^
	competition (*n* = 7)	7.2 ± 1.6 (5.5–10.3)	11.8 ± 2.4 (8.8–15.4)	0.001 ^a^
	training vs. competition *p*-value	0.428 ^a^	<0.001 ^a^	
SAA_adj_ (mg/L) median, IQR (range)	training (*n* = 8)	0.60, 0.29–0.67 (0.23–6.23)	0.63, 0.44–0.76 (0.33–6.67)	0.077 ^c^
	competition (*n* = 7)	0.57, 0.41–0.84 (0.30–3.90)	2.40, 0.75–7.19 (0.35–7.30)	0.023 ^c^
	training vs. competition *p*-value	0.484 ^b^	0.025 ^b^	

^a^ paired Student’s *t*-test; ^b^ Wilcoxon signed rank; ^c^ sign test; Abbreviations: SD, standard deviation; IQR, interquartile range.

**Table 3:** Other hematological and serum biochemical parameters before and after the effort (training and competition).

Parameter Unit	Type of Effort	Time of Blood Collection (mean ± SD (range))		Before vs. After *p*-Value ^a^
		Before Effort	After Effort	
RBC (10^12^/L)	training (*n* = 8)	8.5 ± 1.0 (7.3–10.1)	9.0 ± 1.1 (7.8–10.9)	<0.05
	competition (*n* = 7)	8.8 ± 1.2 (7.0–10.0)	9.5 ± 1.3 (7.6–10.7)	
HGB (mmol/L)	training (*n* = 8)	7.8 ± 0.9 (6.9–9.6)	8.4 ± 1.1 (7.0–10.4)	<0.01
	competition (*n* = 7)	7.4 ± 1.0 (5.8–8.9)	8.1 ± 0.8 (6.8–9.1)	
PCV (l/l)	training (*n* = 8)	37.6 ± 4.6 (32.9–46.4)	40.0 ± 5.0 (34.4–48.6)	≤0.01
	competition (*n* = 7)	38.8 ± 5.4 (30.5–45.2)	42.2 ± 5.7 (33.4–49.1)	
TSP (g/L)	training (*n* = 8)	63.6 ± 4.5 (57–71)	67.1 ± 3.6 (61–72)	≤0.001
	competition (*n* = 7)	64.3 ± 2.9 (61–69)	67.9 ± 4.0 (64–75)	
AST (U/L)	training (*n* = 8)	278.9 ± 28.1 (231–316)	298.7 ± 27.2 (258–328)	<0.05
	competition (*n* = 7)	280.5 ± 37.0 (247–343)	303.2 ± 35.5 (246–347)	
CPK (U/L)	training (*n* = 8)	289.6 ± 108.6 (151–429)	369.4 ± 104.3 (225–512)	<0.05
	competition (*n* = 7)	321.9 ± 141.9 (195–548)	488.7 ± 181.0 (200–774)	

^a^ paired-sample Student’s *t*-test.

## Abbreviations

APR	Acute Phase Response
APPs	Acute Phase Proteins
AST	Aspartate aminotransferase
CPK	Creatine phosphokinase
HGB	Hemoglobin concentration
IL-1	Interleukin 1
IL-6	Interleukin 6
PCV	Packed cell volume
RBC	Red blood cell count
SAA	Serum amyloid A
SAAadj	Serum amyloid A adjusted
TSP	Total serum protein concentration
TSP_1_	Total serum protein level after exertion
TSP_0_	Total serum protein level before exertion
TNF-α	Tumor necrosis factor α
WBC	White blood cell count
